# Supplementary material for: Elevated Glycated Haemoglobin (HbA1c) Is Associated with an Increased Risk of Pancreatic Ductal Adenocarcinoma: A UK Biobank Cohort Study
Source: Cancers (Basel). 2023 Aug 13;15(16):4078. doi: 10.3390/cancers15164078 (PMC10452109; doi:10.3390/cancers15164078)
Supplement: Supplementary file 1 [file cancers-15-04078-s001.zip › Supplementary tables S1 and S2.pdf]

Supplementary Tables

Table S1. Characteristics of study participants at baseline stratified by diabetes mellitus status at enrolment to UK Biobank.

Participant characteristics	No diabetes mellitus on enrolment	Diabetes mellitus on enrolment	Total	*P* value
**Number of Participants, n (%)**	473,264 (94.7%)	26,540 (5.3%)	499,804	<0.001[b]
**Age at attendance (years)[a]**	56.4 (8.1)	59.5 (7.2)	56.5 (8.1)	<0.001[b]
**Men, n (%)**	211,583 (44.7%)	16,095 (60.6%)	227,678 (45.6%)	<0.001[c]
**Glycated haemoglobin (HbA1c) category (mmol/mol), n (%)**				<0.001[c]
<42mmol/mol	420,789 (95.6%)	5,208 (21.3%)	425,997 (91.7%)	
42-47 mmol/mol	15,852 (3.6%)	5,186 (21.2%)	21,038 (4.5%)	
≥48mmol/mol	3,360 (0.8%)	14,038 (57.5%)	17,398 (3.8%)	
**Body mass index (BMI)[a]**	27.2 (4.6)	31.3 (5.9)	27.4 (4.8)	<0.001[b]
**Body mass index (BMI) category, n (%)**				<0.001[c]
Underweight[d]	2,574 (0.6%)	36 (0.1%)	2,610 (0.5%)	
Normal[d]	156,970 (33.3%)	2,838 (10.8%)	159,808 (32.1%)	
Overweight[d]	202,425 (43.0%)	8,961 (34.2%)	211,386 (42.5%)	
Obese[d]	108,990 (23.1%)	14,381 (54.9%)	123,371 (24.8%)	
**Ethnic background**				<0.038[c]
White	448,453 (95.1%)	23,043 (87.29%)	472,616 (94.6%)	
Mixed	2,759 (0.6%)	175 (0.7%)	2,954 (0.6%)	
Asian or Asian British	7,967 (1.7%)	1,703 (6.5%)	9,879 (2.0%)	

Black or Black British	7,052 (1.5%)	914 (3.5%)	8,058 (1.6%)	
Chinese	1,440 (0.3%)	93 (0.4%)	1,573 (0.3%)	
Other ethnic group	4,103 (0.9%)	470 (1.8%)	4,557 (0.9%)	
**Weight change in past year**				<0.001[c]
Lost weight	68,271 (14.7%)	7,293 (28.0%)	75,564 (15.4%)	
No change	263,115 (56.6%)	11,982 (46.0%)	275,097 (56.0%)	
Gained weight	133,463 (28.7%)	6,775 (26.0%)	140,238 (28.6%)	
**Smoking status, n (%)**				<0.001[c]
Never	260,524 (55.3%)	12,133 (46.1%)	272,657 (54.8%)	
Former	161,263 (34.2%)	11,263 (42.8%)	172,564 (34.7%)	
Current (<20/day)	37,210 (7.9%)	2,046 (7.8%)	39,256 (7.9%)	
Current (≥20/day)	12,524 (2.7%)	906 (3.4%)	13,430 (2.7%)	
**Alcohol consumption, n (%)**				<0.001[c]
Never	35,898 (7.6%)	4,343 (16.4%)	40,241 (8.1%)	
Special Occasions	52,787 (11.2%)	4,964 (18.7%)	57,751 (11.6%)	
One to three times a month	52,470 (11.1%)	3,217 (12.1%)	55,687 (11.2%)	
Once or twice a week	122,825 (26.0%)	6,114 (23.1%)	128,939 (25.8%)	
Three or four times a week	111,157 (23.5%)	4,057 (15.3%)	115,214 (23.1%)	
Daily	97,724 (20.7%)	3,800 (14.3%)	101,524 (20.3%)	
**Processed meat intake, n (%)**				<0.001[c]
Never	44,622 (9.5%)	2,007 (7.6%)	46,629 (9.4%)	
Less than once a week	145,167 (30.7%)	6,760 (25.6%)	151,927 (30.5%)	
Once a week	137,846 (29.2%)	7,722 (29.2%)	145,568 (29.2%)	
2-4 times/week	126,339 (26.8%)	8,526 (32.3%)	134,865 (27.1%)	

5 or more times per week	18,259 (3.9%)	1,398 (5.3%)	19,657 (3.9%)	

[a]Mean average (standard deviation)

[b]Student's t test for continuous data

[c]χ^2 test for categorical variables

[d]Adjusted for variations in ethnic-specific variations. Underweight = <18.5kg/m^2, normal 18.5-24.9kg/m2 (18.5 - 22.9kg/m2 in Asian population), overweight = 25.0 - 29.9kg/m2 (23-27.4kg/m2 in Asian population), obese = ≥30.0kg/m2 (27.5kg/m2 in Asian population)

Table S2. Univariable risk of incident pancreatic ductal adenocarcinoma according to glycated haemoglobin, body mass index, weight change compared to one year ago, smoking status, alcohol consumption, processed meat intake, age and sex in subjects with and without diabetes mellitus at enrolment to UK Biobank[a].

Variable	Diabetes on enrolment	Participants (n)	Univariable Hazard Ratio (95% CI)	*P* value	Proportional Hazards assumption
**Glycated haemoglobin (HbA1c)**					
<42mmol/mol	No	420,789	1 (Reference) (N/A[b])		<0.001
42-47mmol/mol		15,852	2.07 (1.62 - 2.66) (N/A[b])	<0.001	
≥48mmol/mol		3,360	2.82 (1.79 - 4.44) (N/A[b])	<0.001	
**Body mass index (BMI)**					
Underweight[c]	No	2,576	0.67 (0.21 - 2.08)	0.615	0.7703
Normal[c]		156,948	1 (Reference)		
Overweight[c]		202,435	1.22 (1.05 - 1.41)	0.01	
Obese[c]		109,028	1.50 (1.27 - 1.76)	<0.001	
**Ethnic background**					
White	No	448,453	1 (Reference)		0.2644
Mixed		2,759	0.82 (0.34 – 1.97)	0.651	
Asian or Asian British		7,967	0.40 (0.19 – 0.84)	0.015	
Black or Black British		7,052	0.58 (0.30 – 1.11)	0.104	
Chinese		1,440	0.93 (0.30 – 2.90)	0.904	
Other ethnic group		4,103	0.79 (0.38 – 1.66)	0.535	
**Weight change compared to one year ago[d]**					

Lost weight	No	68,292	0.95 (0.79 - 1.13) (N/A[b])	0.561	0.032
No change		263,118	1 (Reference) (N/A[b])		
Gained weight		133,465	0.85 (0.74 - 0.98) (N/A[b])	0.03	
**Smoking status**					
Never	No	260,506	1 (Reference)		0.1116
Former		161,339	1.29 (1.13 - 1.48)	<0.001	
Current (<20/day)		37,214	1.57 (1.28 - 1.94)	<0.001	
Current (≥20/day)		12,526	2.63 (2.00 - 3.46)	<0.001	
**Alcohol consumption**					
Never	No	35,904	1 (Reference)		0.2965
Special Occasions		52,799	0.91 (0.68 - 1.20)	0.502	
One to three times a month		52,471	0.65 (0.48 - 0.88)	0.005	
Once or twice a week		122,823	0.87 (0.68 - 1.12)	0.281	
Three or four times a week		111,158	0.85 (0.66 - 1.10)	0.216	
Daily		97,734	1.17 (0.92 - 1.50)	0.197	
**Processed meat intake, n (%)**					
Never	No	44,616	1 (Reference)		0.148
Less than once a week		145,168	1.25 (0.97 - 1.62)	0.079	
Once a week		137,857	1.32 (1.02 - 1.70)	0.036	
2-4 times/week		126,356	1.54 (1.19 - 1.98)	0.001	
5 or more times per week		18,259	1.53 (1.05 - 2.22)	0.025	
**Age**	No	473,264	1.10 (1.09 - 1.11)	<0.001	0.3219
**Sex**					
Female	No	261,677	1 (Reference)		0.5014

Male		211,617	1.33 (1.18 - 1.50)	<0.001	
**Glycated haemoglobin (HbA1c)**					
<42mmol/mol	Yes	5,208	1 (Reference)		0.716
42-47mmol/mol		5,186	1.38 (0.72 - 2.62)	0.33	
≥48mmol/mol		14,038	1.83 (1.07 - 3.13)	0.028	
**Body mass index (BMI)**					
Underweight[c]	Yes	40	6.43 (0.85 - 48.9)	0.072	0.1134
Normal[c]		3,116	1 (Reference)		
Overweight[c]		9,406	1.08 (0.59 - 1.95)	0.811	
Obese[c]		14,664	0.87 (0.49 - 1.55)	0.638	
**Ethnic background**					
White	Yes	23,043	1 (Reference)		0.3887
Mixed		175	1.11 (0.16 – 7.95)	0.917	
Asian or Asian British		1,703	0.23 (0.06 – 0.92)	0.037	
Black or Black British		914	0.64 (0.20 – 2.00)	0.440	
Chinese		93	2.07 (0.29 – 14.91)	0.469	
Other ethnic group		470	0.82 (0.20 – 3.30)	0.776	
**Weight change compared to 1 year ago[d]**					
Lost weight	Yes	7,437	1.37 (0.92 - 2.04)	0.122	0.0915
No change		12,577	1 (Reference)		
Gained weight		7,035	0.84 (0.52 - 1.35)	0.471	
**Smoking status**					

Never	Yes	12,606	1 (Reference)		0.8642
Former		11,638	1.50 (1.01 - 2.23)	0.043	
Current (<20/day)		2,151	2.17 (1.21 - 3.90)	0.01	
Current (≥20/day)		965	2.41 (1.08 - 5.36)	0.031	
**Alcohol consumption**					
Never	Yes	4,425	1 (Reference) (N/A[b])		0.0483
Special Occasions		5,079	1.12 (0.63 - 2.01) (N/A[b])	0.702	
One to three times a month		3,295	0.73 (0.35 - 1.52) (N/A[b])	0.396	
Once or twice a week		6,363	0.87 (0.48 - 1.56) (N/A[b])	0.635	
Three or four times a week		4,278	0.99 (0.53 - 1.86) (N/A[b])	0.983	
Daily		4,069	1.42 (0.79 - 2.55) (N/A[b])	0.244	
**Processed meat intake, n (%)**					
Never	Yes	2,080	1 (Reference)		0.2521
Less than once a week		7,051	2.97 (0.91 - 9.72)	0.072	
Once a week		8,013	3.57 (1.11 - 11.54)	0.033	
2-4 times/week		8,841	3.27 (1.01 - 10.56)	0.048	
5 or more times per week		1,398	5.39 (1.50 - 19.33)	0.01	
**Age**	Yes	26,540	1.07 (1.04 - 1.10)	<0.001	0.3303
**Sex**					
Female	Yes	10,936	1 (Reference)		0.2159
Male		16,621	1.40 (0.96 - 2.03)	0.079	

[a]Participants were asked at enrolment if they had ever been diagnosed with diabetes by a doctor or indicated they were taking medication for diabetes

[b]N/A due to violation of proportional hazards assumption

[c]Adjusted for variations in ethnic-specific variations. Underweight = <18.5mkg/m2, normal 18.5-24.9kg/m2 (18.5 - 22.9kg/m2 in Asian population), overweight = 25.0 - 29.9kg/m2 (23-27.4kg/m2 in Asian population), obese = ≥30.0kg/m2 (27.5kg/m2 in Asian population)

[d]Weight change compared to 1 year ago - participants were asked if they had either lost weight, gained weight or had no change compared to one year prior to enrolment, with the latter as the reference range
